# Osteoporosis and Apical Periodontitis Prevalence: A Systematic Review

**DOI:** 10.3390/dj12080272

**Published:** 2024-08-22

**Authors:** Natália Pestana de Vasconcelos, Isabel Silva Martins, Américo Santos Afonso, Ana Cristina Braga, Irene Pina-Vaz

**Affiliations:** 1 Health Science Faculty, University Fernando Pessoa, 4200-150 Porto, Portugal; nvasc@ufp.edu.pt (N.P.d.V.); alexmar@ufp.edu.pt (I.S.M.); 2 CINTESIS, Faculty of Medicine, University of Porto, 4200-319 Porto, Portugal; 3 Faculty of Dental Medicine, University of Porto, 4200-393 Porto, Portugal; aafonso@fmd.up.pt; 4 ALGORITMI Research Centre, LASI, University of Minho, 4710-057 Braga, Portugal; acb@dps.uminho.pt; 5 CINTESIS@RISE, MEDCIDS, Faculty of Medicine, University of Porto, 4200-450 Porto, Portugal

**Keywords:** bisphosphonates, bone density, endodontics, panoramic radiography, apical periodontitis

## Abstract

Osteoporosis is a common systemic bone disorder in the elderly, characterized by low bone mineral density and deterioration of bone structure. Apical periodontitis is an inflammatory response to the microbial infection of root canals, typically characterized by apical bone destruction surrounding the tooth’s apex. This systematic review aimed to determine if osteoporosis affects the prevalence of apical periodontitis in adults. PRISMA guidelines have been followed. It included randomized clinical trials, cross-sectional, cohort, and case-control studies, and excluded non-relevant investigations and various secondary sources. A comprehensive search was performed in PubMed, Scopus, and Web of Science, until 13 March 2024. The Newcastle–Ottawa Scale was used to assess the quality of the three selected studies: two cross-sectional studies and one case-control study. One investigation only included post-menopausal women recruited at a dental university clinic, the other integrated data from the total hospital patients’ population, and the third selected patients referred to the university dental clinic from the university hospital. The findings varied: one study noted a marginal association between low bone mineral density and apical periodontitis, another found a significant association, and the third, with the lowest risk of bias, reported no link. The main limitations were the scarcity of eligible studies and their overall quality. The review was registered in the PROSPERO database (CRD42024523705), applied strict inclusion criteria and thorough searches by experienced and independent reviewers. There is no strong evidence that adult individuals with osteoporosis have a higher probability of developing apical periodontitis. However, clinicians should remain cautious of osteoporosis’s potential impact on apical periodontitis development.

## 1. Introduction

Osteoporosis (OP) is a frequent systemic bone disturbance in the elderly, often diagnosed after experiencing fracture, characterized by low bone mineral density and deterioration of bone structure [1]. A recent systematic review confirmed that OP and osteopenia represent a significant worldwide public health issue [2]. This review, based on the World Health Organization diagnostic criteria, found global prevalences of OP and osteopenia of 19.7% and 40.4%, respectively. The prevalence rates are higher in developing countries (22.1%) compared to developed countries (14.5%) [2]. Regarding sex-specificity prevalence, OP and osteopenia affect 10.6% and 44.8% of males, respectively. Among females, the rates are 24.8% for OP and 39.4% for osteopenia, with post-menopausal women showing higher prevalences (27.4% and 42.1%) [2]. Geographically, Africa exhibits the highest prevalence, while Oceania had the lowest. OP prevalence rises with age, reaching 20.5% for individuals over 50, exhibiting the most significant prevalence of 40.8% observed in the group of 80–89 years [2]. Health education levels, different medical care systems, and levels of urbanization were also associated with a higher risk of OP [2].

The balance between osteoclastic and osteoblastic activity is essential in bone repair, and disruptions in this balance contribute to OP [3]. Failing to achieve a normal peak bone mass or experiencing an accelerated bone loss rate can also contribute to the development of OP. The hormonal changes during menopause, especially the decrease in estrogen production and the increase in certain hormones like pituitary follicle-stimulating hormones (FSH), lead to accelerated bone loss, altered calcium metabolism, and increased risk of OP [4,5]. OP is, thus, a multifactorial disease in which age, gender, calcium and vitamin D intake, exercise routines, hereditary factors, and the presence of essential arterial hypertension can all be implicated [6,7] OP has two main types: primary OP, which is typically linked to ageing or reduced levels of sex hormones, and secondary OP, which is associated with various medical conditions, medications like glucocorticoids and anti-epileptics, and lifestyle factors [8,9]. Bisphosphonates (BPs) and denosumab are commonly used to treat OP. They have immunomodulatory effects and inhibit the activity of cells like osteoclasts, which are responsible for bone tissue resorption. Thus, these medications help prevent further bone loss and improve bone density [8].

Apical periodontitis (AP) is an inflammatory response, essentially due to the presence of pathogens and their toxins in the root canal system [10]. It can arise from various primary factors, including the progression of dental caries, trauma, or operative dental procedures, but its main cause is pulp infection. Other etiological factors include fractures of the tooth structure, iatrogenic procedures, or any circumstances that allow bacteria to penetrate the pulpal tissues [10]. The bacteria and the toxins they release, as well as other substances, like immunological agents, lead to an inflammatory reaction in the periradicular ligament, causing the progression of the periradicular inflammation [10,11,12]. This inflammatory process involves the recruitment of inflammatory cells with enzyme release, interleukin production, and activation of bone resorptive cells, resulting in periapical alveolar bone resorption [4,13]. AP typically manifests as a chronic, asymptomatic condition marked by a radiolucent lesion surrounding the tooth’s apex. It is one of the most prevalent oral infections alongside dental caries. It is estimated that half of the world’s adult population has at least one tooth affected by AP [14]. While acute AP can present as a symptomatic inflammation, chronic AP is more commonly evaluated in prevalence studies due to the easily recognizable radiographic lesions that facilitate comparison across different populations. Even though periapical infections elicit local tissue responses to contain the spread of infectious agents, AP is not just a local concern; substantial evidence suggests that chronic AP may contribute to systemic inflammation [15]. Recent studies suggest a high global prevalence of AP in the adult population, with a notable increase over the last decade [16]. Moreover, it was noted that a systemic proinflammatory status can impact the repair of AP following endodontic treatment [13,14].

Advancements in endodontics have prompted researchers to further investigate the impact of overall health conditions on endodontic infection prevalence. The common etiologic factors of OP and AP complicate efforts to conclusively determine a potential link. Both OP and chronic AP involve inflammation-induced osteolysis. In OP, reduced bone density results from a disproportion between bone formation and bone degradation, with systemic influences like estrogen affecting the equilibrium of bone metabolism [13]. Evidence suggests that the inflammation-induced bone loss in OP may worsen chronic AP and vice versa [17]. Studies in animals have found that estrogen-deficient rats experienced a higher rate of bone loss in periapical lesions [18]. Additionally, BPs appeared to slow the progression of AP in patients with OP [19,20,21]. Nevertheless, the relationship between OP and AP has not received widespread discussion. In fact, one of the few recent systematic reviews assessing dental alterations, including the prevalence of periradicular radiolucencies, primarily focused on patients under BP therapy [22]. Therefore, the present investigation systematically reviewed the existing literature to assess the potential link between OP and the prevalence of AP.

## 2. Materials and Methods

### 2.1. Protocol and Registration

In line with the 2020 Preferred Reporting Items for Systematic Reviews and Meta-Analyses (PRISMA) guidelines, this systematic review focused on the possible association between OP and AP prevalence [23]. The protocol was previously prepared and registered in the Prospective Register of Systematic Reviews (PROSPERO) database (CRD42024523705).

### 2.2. Eligibility Criteria

Consistent with the Population–Intervention–Comparator–Outcomes (PICO) structure, the question elements were addressed as detailed below:

→ Population/participants: Adult individuals (≥18 years old)
→ Intervention(s), exposure(s): Patients with OP
→ Comparator(s)/control: Healthy individuals (without OP)
→ Outcome: Prevalence of AP associated with or without root-filled teeth in patients diagnosed with OP

The research question addressed was: Does OP affect the prevalence of AP in adults?

→ Inclusion criteria: Studies reporting the prevalence of AP from adult individuals with OP and healthy controls, in randomized clinical trials, cross-sectional, cohort, and case-control studies.
→ Exclusion criteria: Animal or laboratory investigations, studies not including a control group of healthy individuals, studies not reporting AP prevalence. Studies that did not address the specific research question were excluded. Repeated findings, meta-analyses, scoping, systematic, or narrative reviews, meeting abstracts, case series, and case reports, were excluded.

### 2.3. Search Strategy

The search was conducted on 13 March 2024 on PubMed (Medline), Scopus, and Web of Science. The electronic search combined medical subject heading (MeSH) terms and text words (tw) based on the PICO strategy. The Boolean operators “AND” and “OR” were used to create the search strategy (Table 1). No language and publication date restrictions were applied.

The bibliography of all included papers were also hand-searched. Additionally, other digital repositories such as Google Scholar (the first 100 returns were considered) and OpenGrey were analyzed to select pertinent doctoral dissertations, conference papers, and unpublished manuscripts and uncover other grey literature sources.

The results obtained upon the literature search were imported into the EndNote X9 software (Thomson Reuters, New York, NY, USA), which automatically removed duplicate records.

### 2.4. Selection of the Studies

This systematic review was performed with a two-step screening procedure to select studies for inclusion. In the first stage, two independent reviewers assessed the titles and abstracts of previously identified publications, registering the criteria for exclusion of non-eligible papers. In the second stage, reviewers evaluated the full texts of studies identified as eligible in the initial screening. The lists of pertinent studies were compared, and in case of divergence, a third reviewer determined the eligibility of papers for inclusion. All studies failing to meet the inclusion criteria were excluded from the analysis.

### 2.5. Data Extraction

Two independent examiners performed data extraction. All extracted data were compiled and organized into tables using Microsoft Excel v.16.43 (Microsoft Corporation, Redmond, WA, USA). The following information was extracted and registered from each included study: name of the first author, year published, type of study design, total number of participants with age distribution, population characteristics, investigated outcomes of interest, diagnostic criteria for AP, and main results. A third reviewer resolved any disagreements or uncertainties.

### 2.6. Quality Assessment

The risk of bias assessment for case-control and cross-sectional studies was conducted using the Newcastle–Ottawa Scale (NOS) and its adaptation for cross-sectional studies, respectively [13]. Two independent reviewers used the NOS star rating system to critically appraise each included study based on three domains: selection, comparability of the groups, and outcome assessment. Specific items were evaluated in each domain, and each criterion corresponded to a star. The total number of stars given to each study reflected its overall quality. Studies were categorized as having high quality (7–9 stars), moderate quality (4–6 stars), or low quality (0–3 stars) based on the number of stars received. Moreover, studies with 7–9 stars were considered “Good” (low risk of bias), those with 4–6 stars were considered “Fair” (moderate risk of bias), and those with fewer than 3 stars were marked as “Poor” (high risk of bias).

Any disagreements during the assessment process were resolved through discussion between the two reviewers, and a third reviewer was involved if necessary. This approach ensured a thorough and standardized evaluation of the quality of the included studies, enhancing the reliability of the review’s findings.

## 3. Results

### 3.1. Literature Search Process

**Figure 1:**
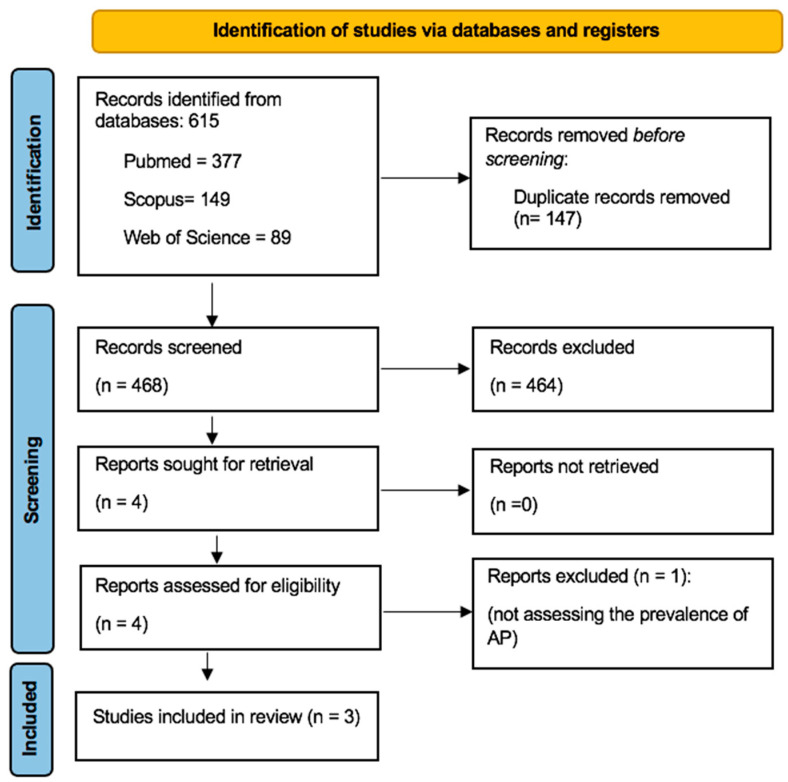
PRISMA flow diagram of the study search and identification of relevant studies.

After searching the three databases, 615 articles (377 from PubMed, 149 from Scopus, and 89 from Web of Science) about the prevalence of AP among patients with OP were identified, of which 147 duplicates were excluded. The remaining 468 relevant titles and abstracts were reviewed and screened based on the established selection criteria, leading to the exclusion of 464 articles. After a full-text review of the remaining four articles, one was excluded [24]. The aim of this retrospective investigation was to compare the outcome of the non-surgical root canal treatment in patients receiving intravenous zolendronate. All the investigated patients had AP and were referred for root canal treatment. In this context, the study primarily focused on the progression of AP after endodontic treatment, rather than directly assessing the influence of OP on the prevalence of AP. Finally, three studies met the criteria for quality assessment and were included in this systematic review (Figure 1).

### 3.2. Characteristics of the Included Studies

Table 2 lists the main characteristics of the three included investigations: two cross-sectional studies [4,25] and one case-control study [5]. All the studies were written in English and published between 2015 and 2022. Two of them were conducted in Europe, one in Italy [5], and another in Spain [4], and one study was performed in the USA [25]. In total, these studies enrolled 1,645,180 adults. The sample size of the included studies ranged from 75 [4] to 1,644,953 participants [25]. Two studies comprised men and women [5,25], while one included only post-menopausal women [4]. The approximate age range was 62.26 years [4,5], but one of the studies did not specify the participant’s age [25].

All the studies diagnosed AP by panoramic radiography and, in some cases, complemented it with periapical radiography [5]. Only one of the studies used the periodontitis apical index (PAI) to evaluate AP [5]. The other two studies used other criteria related to the periodontal ligament width to assess periapical status: one [4] defined AP according to the criteria described by Halse and Molven [26], and the other [25] defined AP as radiographic evidence of apical rarefying osteitis.

The three studies analyzed the association between the prevalence of AP and OP. Two studies also investigated the association of the different medications for OP with the prevalence of AP [5,25]. One study [5] assessed the prevalence of AP in root-filled teeth and the quality of the root canal filling and coronal restoration, considering its presence as the post-therapy persistence of AP. In one study, OP was defined according to the International Classification of Diseases 9th revision (ICD-9) code 733 and the International Classification of Diseases 10th revision (ICD-10) code M81. The other two studies defined OP according to the World Health Organization criteria for bone mineral density and excluded participants with other systemic factors of bone remodeling [4,5].

### 3.3. Main Findings

#### 3.3.1. AP Prevalence in OP Patients

AP prevalence was defined as the number of individuals with at least one tooth with a periapical lesion. Two studies [4,25] found significant differences in the prevalence of AP between OP patients and controls, while Cadoni et al. [5] found none.

López-López et al. [4] observed a marginally significant link between low bone mineral density and AP, with no significant differences in age, number of teeth, number of root-filled teeth, or number of teeth with coronal restorations.

Katz and Rotstein [25] reported a higher prevalence of AP in OP patients, especially those not treated with BPs, highlighting the impact of OP medications. Specifically, patients on risedronate exhibited lower AP prevalence than those on alendronate.

#### 3.3.2. The Impact of OP Medications on the Prevalence of AP

In one study [25], patients treated with BPs presented a lower OR for AP than non-treated patients: 2.35 versus 3.52, respectively. Patients with OP treated with any type of BP showed a 1.25% AP prevalence compared with 0.52% in the general patient population of the hospital. Additionally, patients treated with risedronate, a more potent BP, showed a lower prevalence of AP than patients treated with alendronate. Patients treated with alendronate showed an OR of 1.6 for AP. In turn, patients treated with risedronate showed an OR of 1.34 for AP. In sum, Katz and Rotstein [25] concluded that the prevalence of AP was significantly higher in OP patients, with an especially marked reduction when risedronate was used for OP treatment.

Conversely, Cadoni et al.’s case-control study [5] reported no association between OP and AP prevalence, irrespective of the condition: untreated or treated with therapeutic agents like BPs and denosumab. AP prevalence was 42.1% in the OP group, compared with 47.4% in the control group (*p* = 0.62). Regarding AP prevalence among the three OP subgroups, no significant differences existed between individuals under pharmacological treatment and those not medicated for the disease (*p* = 0.61). Patients in the denosumab group showed the highest AP prevalence (66.7%), followed by those previously treated with BPs and then denosumab (63.6%), those not treated (36.0%), and those treated with BPs alone (32.3%) (*p* = 0.11). The number of teeth with AP was similar between all groups. A higher PAI was observed in the control group (3.04) compared to the OP group (2.79) (*p* = 0.36). A multivariate logistic regression analysis was conducted to investigate the influence of gender, age, medications, duration of medication use, smoking status, and the number of teeth on AP prevalence. Considering all these factors as covariates, individuals undergoing treatment with denosumab showed a higher risk for AP (OR = 1.83; CI 95% = 1.15–3.37; *p* = 0.03). None of the other variables showed an association with AP.

#### 3.3.3. Progression of AP

In Cadoni et al.’s study [5], AP was notably more prevalent in root-filled teeth than non-treated teeth in patients with OP, while there was no significant difference between treated and non-treated teeth in the control group (*p* = 0.03). The quality of endodontic treatment and coronal restoration in root-filled teeth with AP was considered similar in both OP and control groups. Finally, the number of decayed, missing, and filled teeth was significantly lower in the OP group compared with the control group, with averages of 22.25 teeth (*p* < 0.01) versus 24.57 teeth (*p* = 0.03), respectively.

#### 3.3.4. Quality of the Studies

Quality was assessed for each study. NOS-based results demonstrated that Cadoni et al.’s [5] case-control study was of “Good” quality, with 7 stars, while Katz and Rotstein [25] and López-López et al.’s [4] cross-control studies were of “Fair” quality, with 4 and 5 stars, respectively (Supplementary Materials Tables S1 and S2). None of the studies justified the sample size or reported a blinded assessment of medical history or radiographic exam evaluation.

## 4. Discussion

This systematic review explored the potential relationship between OP and AP, focusing on whether OP influences the prevalence of AP in adults. An extensive literature search and the removal of duplicates and non-qualifying records resulted in the selection of three studies [4,5,25] from an initial 615, consisting of two cross-sectional studies [4,25] and one case-control study [5]. The inclusion criteria targeted clinical evidence applicable to human populations, favoring randomized trials, cohort studies, case-control studies, and cross-sectional studies for their respective strengths in establishing causal relationships, assessing outcomes, and capturing prevalence [27]. However, due to study heterogeneity, a qualitative synthesis was used instead of a meta-analysis.

The findings initially suggested that OP might increase AP prevalence, with two of the three studies showing an association. Nonetheless, a critical appraisal using the Newcastle–Ottawa Scale (NOS) revealed methodological flaws, with the referred studies by Katz and Rotstein [25] and López-López et al. [4] rated as “Fair”. Notable weaknesses included sample representativeness and size, impacting the reliability of the conclusions. On the other hand, Katz and Rotstein’s study [25] had a large and diverse sample size but lacked clarity on the composition of the control group.

In Cadoni et al.’s investigation [5], rated as “Good,” the authors found no association between OP and AP prevalence. Although the differences between OP groups (treated with BPs, with denosumab, or not treated) were not statistically significant, patients from the denosumab group presented a higher risk for AP. Surprisingly, a higher PAI score was found in the control group compared to the OP patients, regardless of the varying treatments or unused medication. The selection of cases and controls was considered adequate. Controls were randomly selected among patients of the dental clinic from the same university as the cases. Nevertheless, while the diagnosis of OP patients (cases) was based on bone mineral density, controls were defined as not having a history of OP. Age, sex, smoking habits, and socioeconomic status were matched between cases and controls. However, the rate of exposure was unclear and the representativeness of the sample was not precisely described, nor was its size justified. In OP patients, root-filled teeth were significantly more frequently affected by AP than non-treated teeth, suggesting a lower success rate in post-therapy periapical healing. No such difference was noted in the control group. Hence, endodontic treatment and restoration were considered of comparable quality. Supporting these findings in systemically compromised patients, Jakovljevic et al. [13] highlighted a significant increase in AP occurrence in root-filled teeth associated among individuals with gastrointestinal diseases compared to healthy controls. The authors stressed the importance of dentists being aware of these findings when treating patients with systemic disorders, particularly those related to low-grade inflammation, such as AP.

The three studies used panoramic digital images to assess whether AP was present or absent in OP patients. Panoramic radiography is a practical tool employed in epidemiological studies [28]. Although it can potentially underestimate periapical lesions comparing to periapical X-ray, numerous investigations have utilized this method effectively [29,30,31]. Additionally, it has been suggested that panoramic radiography could be a valuable tool for confirming AP in individuals with low bone mineral density [32] while other imageology techniques, such as cone-beam computed tomography (CBCT) were questioned [33].

Although all the selected studies employed panoramic radiography for AP prevalence assessment, they varied in their evaluation criteria. In Katz and Rotstein’s investigation [25], the presence of AP was based on a query and defined as a radiographic apical radiolucency, not extensively specified. López-López et al. [4] reported that three investigators assessed digital panoramic radiographs. Intra- and inter-observer calibration was conducted to ensure an accurate and consistent interpretation. AP was defined as a periodontal ligament space larger than the normal width according to the criteria previously described by Halse and Molven [26]. Finally, Cadoni et al. [5] based their outcome assessment on AP records observed by four trained endodontists, analyzing PAI scores [34] in panoramic and selective periapical radiographs. The PAI index is widely utilized in epidemiological and clinical studies to determine the prevalence of AP [30,35,36].

BPs and denosumab are antiresorptive and immunomodulatory medications that represent the current treatment of choice for severe cases of OP [37]. Considering the pivotal role of osteoclasts in bone remodeling, BPs could potentially influence the healing of periapical lesions. Katz and Rotstein [25] found a lower incidence of AP in patients treated with BPs and with differences between alendronate and risedronate groups. These findings agree with previous studies that showed that BPs effectively inhibit AP progression in OP patients [17,18,19,20,21,38]. On the other hand, Cadoni et al.’s study [5] did not find significative differences in AP prevalence between the control (without OP) and the study group of OP patients (not treated, treated with BP, treated with denosumab). AP prevalence was lowest in patients receiving BPs, although this difference was not statistically significant. These findings align with the results reported by Katz and Rotstein [25].

In the present investigation, different scenarios of OP patients were evaluated: not treated [5,25], treated with BPs, such as alendronate or risedronate [5,25], and treated with a monoclonal antibody, such as denosumab [5]. López-López et al. [4] subdivided the osteoporotic patients into osteogenic and osteoporotic, based on mineral bone density assessed by densitometry. OP classification varied across studies. One study defined OP based on the ICD-10 code M81 [5], while others utilized the World Health Organization criteria for bone mineral density [4,5]. Additionally, only one study [5] implemented inclusion criteria based on the diagnosis of primary OP, aiming to minimize potential confounding variables.

The main limitations of the present systematic review include the limited number of primary investigations selected and the fact that two [4,25] out of the three selected studies were rated as “Fair” in terms of quality. Concerns arise from the unexplained sample size, lack of blinding assessment, unmatched study groups, and unadjusted confounding factors related to AP progression. Additionally, the quality of the root canal fillings and coronal restorations was only evaluated in Cadoni’s study [5]. On the other hand, the strengths of this systematic review include (1) having a registered a priori protocol in the PROSPERO database; (2) applying strict eligibility criteria, having excluded studies without a group control with healthy individuals or not directly assessing AP prevalence; (3) conducting thorough literature searches across three electronic databases, with no time and language restrictions, by two experienced and independent reviewers; and (4) critical appraisal of included studies using the NOS also conducted by two reviewers independently. Despite having strictly followed the PRISMA guidelines for systematic reviews, our findings should be cautiously interpreted due to the heterogeneity between the included studies.

Although an association between OP and AP cannot be clearly determined, this relation can be plausible. Biological experimentations conducted in animal models support a possible association between OP and AP [18,19,20,21,39]. Additionally, animal studies have explored innovative approaches to OP treatment, aiming to avoid the well-known side-effects associated with conventional BP treatments [40]. The present review is important to highlight the challenges of bone healing in OP patients and its potential implications for oral health. AP radiolucency’s size or PAI assessment might be masked due to the medications aiming to treat OP. Furthermore, if we consider that OP patients might experience delays in bone healing, the prognosis of endodontic treatment in these individuals may be compromised. Therefore, dentists and other healthcare professionals should be mindful of the potential influence of OP on the development of AP and collaborate with physicians. Clinicians should consider this when planning and managing root canal treatment in OP patients, as they may need to adjust treatment strategies accordingly. Prospective studies with high standards of quality research are needed. These studies would help to better understand the extent of this potential association, enhance novel preventive and treatment strategies for both conditions, and improve global health.

## 5. Conclusions

The present review indicates that OP may have an impact in the prevalence of AP. However, the currently available scientific evidence concerning the possible association between OP and AP is limited. The heterogeneity between the included studies leaves the relationship between these two conditions somewhat uncertain. Further research, particularly high-quality longitudinal studies, is needed to deepen our understanding and guide clinical practice.

## Figures and Tables

**Table 1:** Search strategy.

Database	Search Strategy	Findings
PubMed	#1 (osteoporosis OR bisphosphonates OR menopause)	216,172
	#2 (Endodontics OR Periapical Periodontitis OR Periapical Diseases OR Apical Periodontitis OR Periradicular Lesion OR Periapical Radiolucency OR Radiolucent Periapical Lesion)	65,113
	#1 and #2	377
Scopus	#1 (osteoporosis OR bisphosphonates OR menopause)	249,224
	#2 (Endodontics OR “Periapical Periodontitis” OR “Periapical Diseases” OR “Apical Periodontitis” OR “Periradicular Lesion” OR “Periapical Radiolucency” OR “Radiolucent Periapical Lesion”)	40,924
	#1 and #2	149
Web of Science	#1 (osteoporosis OR bisphosphonates OR menopause)	192,512
	#2 (Endodontics OR “Periapical Periodontitis” OR “Periapical Diseases” OR “Apical Periodontitis” OR “Periradicular Lesion” OR “Periapical Radiolucency” OR “Radiolucent Periapical Lesion”)	27,151
	#1 and #2	89

**Table 2:** The characteristics and main results of studies included in systematic review.

Authors, Year	Study Design	Number of Participants/Age (Mean ± Standard Deviation Range)	Population Characteristics	Investigated Outcomes of Interest	Exposure Evaluation Method/AP Definition	Main Results
Lopez-Lopez et al. 2015 [4]	Cross-sectional study	75 (62.5 ± 1.7)/years Ostoporotic (12) Osteopenic (36) Control (27) Greater than 50 years old	Post-menopausal women recruited at the Dental Clinic of the University of Barcelona, Spain	- Number of teeth present - Number and location of root-filled teeth - Number and location of teeth having coronal restorations - Number and location of teeth having AP	Panoramic radiograph (AP was defined as periodontal ligament space larger than the normal width)	A marginally significant association was evident between low bone mineral density (BMD) and the presence of AP (OR = 1.9; CI 95% = 1.0–3.8; *p* = 0.050)
Katz et al. 2021 [25]	Cross-sectional study	1.644.953 Age not specified	Integrated data from the total hospital patient’s population of the University of Florida (USA) Health Office for the period of 2011–2020 were used	- Prevalence of AP - Prevalence of OP - Prevalence of AP in patients with OP - Prevalence of AP in patients treated with alendronate and risedronate	Panoramic radiograph (AP was defined as radiographic evidence of apical rarefying osteitis)	The prevalence AP was significantly higher in OP patients (OR = 3.36; *p* < 0.0001) OP patients treated with BPs showed a marked reduction in the prevalence of AP (*p* < 0.0001)
Cadoni et al. 2022 [5]	Case-control study	Cases: 76/64.61 ± 8.09 years D (9) BPs (31) BPs + D (11) NM (25) Control: 76/59.67 ± 9.88 years	Patients diagnosed with OP without systemic conditions, referred to the University Dental Clinic from the Departments of Rheumatology and Orthopedics at the Cagliari University Hospital (Italy), from February 2015 to October 2020	- Number of teeth present - Number of caries and AP - Prevalence of AP - Decayed, missing, filled teeth index - Prevalence of AP in root canal-treated teeth - Quality of root-filled teeth and restoration	Periapical radiograph Panoramic radiograph PAI score	Primary OP does not appear to be associated with the prevalence of AP regardless of whether the condition is untreated or treated with therapeutic agents like BPs and denosumab The prevalence of AP was higher in root-filled teeth in the OP group (*p* = 0.03)

## Data Availability

The data presented in this study are available from the corresponding author upon reasonable request.

## Supplementary Materials

The following supporting information can be downloaded at: https://www.mdpi.com/article/10.3390/dj12080272/s1, Table S1: Critical appraisal of included case control study via Newcastle–Ottawa Scale tool [5]; Table S2: Critical appraisal of included cross-sectional study via adapted Newcastle–Ottawa Scale tool [4,25].
