# Supplementary material for: Osteoporosis and Apical Periodontitis Prevalence: A Systematic Review
Source: Dent J (Basel). 2024 Aug 22;12(8):272. doi: 10.3390/dj12080272 (PMC11354161; doi:10.3390/dj12080272)
Supplement: Supplementary file 1 [file dentistry-12-00272-s001.zip › dentistry-3115609-supplementary.pdf]

TABLE S1 Critical appraisal of included case control study via Newcastle-Ottawa Scale tool [5]

NOS - Case-Control study											
**Study (year)**	**Selection Definition**	**Selection Representative**	**Selection Non-respondents**	**Selection Definition control exposure**	**Comparability Appropriate cases and controls**	**Comparability Other Factors**	**Exposure Secure Record**	**Exposure Blinded**	**Exposure Same Methodology**	**Exposure Same Rate**	**Stars / Results**
**Cadoni et al. 2022**	*	sample size not justified	*	*	*	*	*	not reported	*	not reported	7 /Good

TABLE S2 Critical appraisal of included cross-sectional study via adapted Newcastle-Ottawa Scale tool [4,25]

NOS – adapted for Cross-sectional studies

Study (year)	Selection Representative	Selection Non-respondents	Selection Exposure	Comparability Appropriate control	Comparability Other Factors	Outcome Independent	Outcome Record Linkage	Statistical Test	Stars / Results
**Katz et al. 2021**	*	not completely specified	*	*		blind assessment not reported		*	4 / Fair
**Lopez-Lopez et al. 2015**		*	*	*		blind assessment not reported	*	*	5// Fair
